# Using satellite imagery to evaluate precontact Aboriginal foraging habitats in the Australian Western Desert

**DOI:** 10.1038/s41598-021-89642-1

**Published:** 2021-05-25

**Authors:** W. Boone Law, Peter Hiscock, Bertram Ostendorf, Megan Lewis

**Affiliations:** 1 School of Biological Sciences and Environment Institute, University of Adelaide, Adelaide, SA 5005 Australia; 2 Geoscience and Archaeology, Australian Museum, 1 William Street, Sydney, NSW 2010 Australia

**Keywords:** Archaeology, Ecological modelling, Ecology, Behavioural ecology, Ecology, Palaeoecology

## Abstract

Modern satellite imaging offers radical new insights of the challenges and opportunities confronting traditional Aboriginal ecology and land use in Australia’s Western Desert. We model the likely dynamics of historic and precontact desert land use using Earth observation data to identify the distribution of suitable foraging habitats. Suitability was modelled for an ideal environmental scenario, based on satellite observations of maximal water abundance, vegetation greenness, and terrain ruggedness. Our model shows that the highest-ranked foraging habitats do not align with land systems or bioregions that have been used in previous reconstructions of Australian prehistory. We identify impoverished desert areas where unsuitable foraging conditions have likely persisted since early in the last glacial cycle, and in which occupation would always have been rare. These findings lead us to reconsider past patterns of land use and the predicted archaeological signature of earlier desert peoples.

## Introduction

The ‘Western Desert’, as it is colloquially known, is one of the most arid and geographically remote regions of continental Australia. Understanding the dynamics of human ecology in this vast arid region is important to the investigation and interpretation of the continent’s Aboriginal archaeological record. Of Australia’s many desert areas, the Western Desert has been featured prominently in the development of many precontact human ecology models^1–7^. The founding ‘Australian Desert Culture’ model^1^, created to explain human occupation of the Western Desert, was grounded in its use of the ethnographic record to characterise traditional land use practices, and continues to influence research today. But modern research explores desert archaeology in diverse ways, using principles of biogeography, evolutionary biology and human behavioral ecology^8–14^. Those models have enhanced our understanding of traditional forager subsistence and settlement practices, highlighting the complex nature of the Aboriginal land use. However, scale remains an issue in subsequent models, where broad, regional-level environmental characterizations have created low resolution understandings of resource availability and land use, thereby limiting their ability to measure changing land use practices over time and space.

Australian researchers have increasingly employed spatial science methodologies to better portray ecological scenarios to interpret the archaeological past, although the ecological grain of such models has thus far been limited to coarse-scaled, continental-wide representations^13,15–18^. Technological advances in spatial science and environmental remote sensing offer a new level of sophistication to investigate past land use in a more spatially explicit way and at higher resolution than earlier models. With more than a quarter-century of Earth observation imagery available in open access formats, it is easier than ever to access and process massive geospatial and remote sensing datasets into sophisticated models of human ecology. Our research takes advantage of these repositories, utilizing a number of freely available satellite image-derived products to investigate past Western Desert land use, including Geoscience Australia’s^19^ Water Observations from Space (WOfS), the United States Geological Survey’s^20^ Landsat 5 TM Normalized Difference Vegetation Index (NDVI) datacube, and the Japan Aerospace Exploration Agency’s^21^ Advanced Land Observing Satellite’s (ALOS) World 3D 30 m digital surface model. We use these remote sensing products to derive indicators of water accessibility, vegetation greenness, and terrain ruggedness. In combination, these variables are used to build a spatial ecological model that identifies suitable foraging habitats during times of maximum water abundance and above average vegetation health. Our suitability model has implications for how we think about and test for land use histories, which we contextualize with previous ideas of Aboriginal subsistence and settlement in the Western Desert.

## Background and rationale

**Figure 1:**
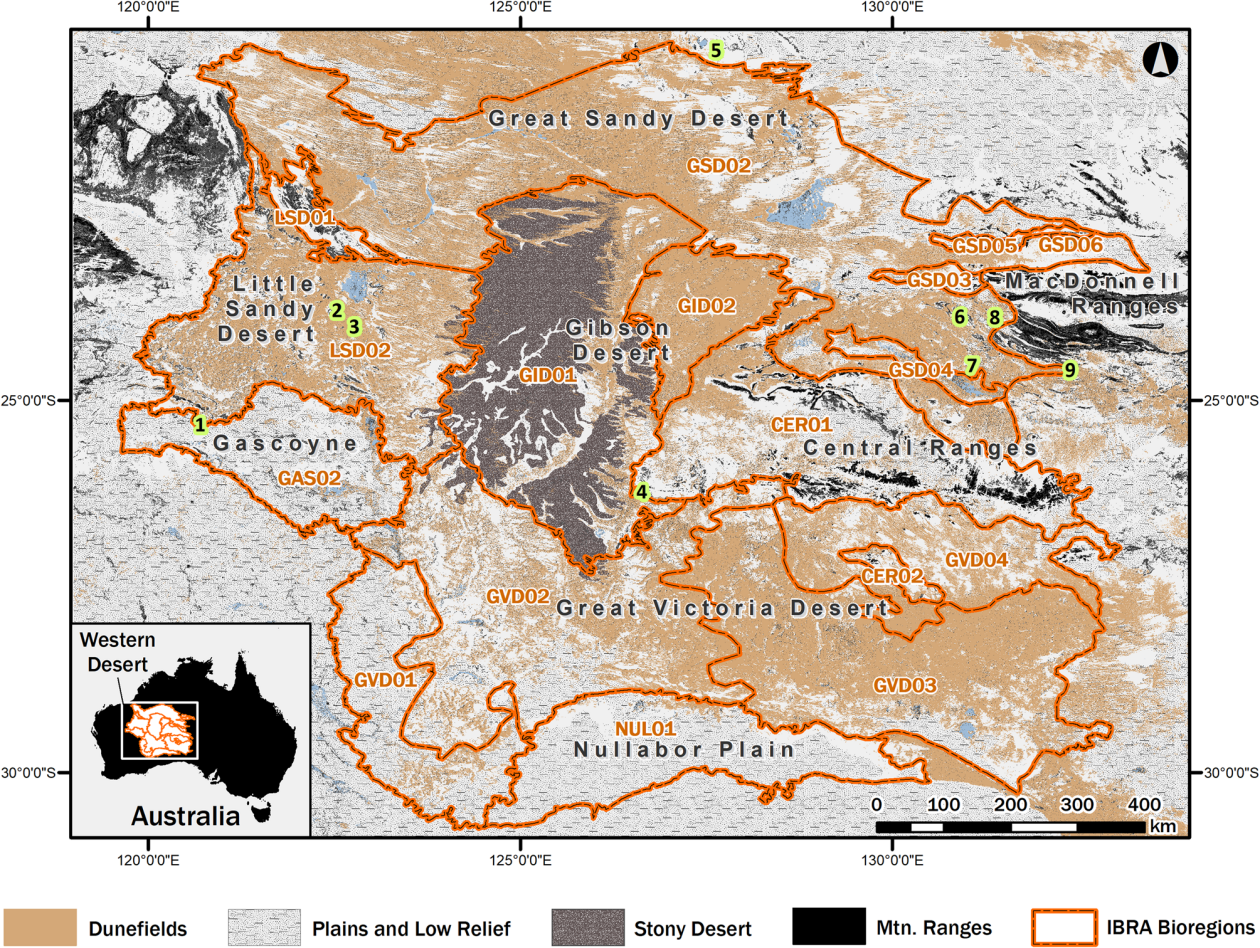
Location of the Western Desert in relation to continental Australia. The bioregional boundaries of the Interim Biogeographic Regionalisation for Australia (IBRA)^23^ subregions are shown in relation to major land systems and desert regions. The code keys to IBRA subregions are: CER01-Central Ranges (Mann-Musgrave), CER02-Central Ranges (Watarru), GAS02-Gascoyne (Carnegie), GID01-Gibson Desert (Lateritic), GID02-Gibson Desert (Dunefield), GSD02-Great Sandy Desert (MacKay), GSD03-Great Sandy Desert (Ehernberg), GSD04-Great Sandy Desert (Amadeus), GSD05-Great Sandy Desert (Lake Bennett), GSD06-Great Sandy Desert (Lake Lewis), GVD01-Great Victoria Desert (Shield), GVD02-Great Victoria Desert (Central), GVD03-Great Victoria Desert (Maralinga), GVD04-Great Victoria Desert (Kintore), LSD01-Little Sandy Desert (Rudall), LSD02-Little Sandy Desert (Trainor), and NUL01-Nullarbor (Carlisle). Excavated rockshelter sites with long archaeological sequences (lime green) include: (1) Karnatukul (Serpent’s Glen), (2) Bushturkey-3, (3) Kaalpi, (4) Puntuntjarpa, (5) Parnkupirti, (6) Puritjarra, (7) Glen Thirsty, (8) Tjungkupu, and (9) Kulpi Mara. Map created in ESRI ArcGIS Desktop 10.5.1 (https://desktop.arcgis.com). Vector layers adapted from Creative Commons datasets (GEODATA Topo 250 K Series 3, Atlas of Australian Soils, and IBRA Version 7 Subregions) available at https://data.gov.au. Montane upland layer based on terrain ruggedness raster (see “Methods” section).

Australia’s ‘Western Desert’ actually encompasses a diverse array of arid landscapes that are often discussed at different spatial scales. At a very generalised level, its prominent geographical areas include the Great Sandy Desert, Little Sandy Desert, Great Victoria Desert, Gibson Desert and the western portions of the Tanami Desert and the Central Ranges (Fig. 1). Alternatively, for analytical purposes, the Western Desert is also divided into 17 subregions under a nationally agreed upon framework known as the Interim Biogeographic Regionalisation for Australia (IBRA)^23^ (Fig. 1). The boundaries of the IBRA subregions are spatially delineated using a combination of climatic, geological, landform, native vegetation and species information, and they are commonly used by the scientific community to summarise and broadly investigate inter-regional biodiversity and ecosystem dynamics across the arid zone^10,18,24–26^.

The greater Western Desert, as defined here, spans nearly 1.1 million km^2^, representing roughly 14.0% of the Australian land mass. Rainfall is seasonally unreliable, with median annual rainfall around 250 mm^27^. Ambient temperatures exceeding 35 °C occur more than 114 days annually and evapotranspiration is very high. The Western Desert is riverless, lacking coordinated drainage or permanent freshwater lakes^26^. Reliable water is largely confined to montane uplands or rocky outcrops, but there are exceptions. Springs, soaks and subterranean chambers are known to occur in palaeodrainage networks hundreds of kilometers from any rocky substrate^28^. In all other landforms, the availability of surface water is short-lived, often in playas^29^, and entirely contingent on rain.

These environmental conditions presented severe challenges for human survival, and our concern is the implications of geographical variation in resource availability in those diverse desert environments for historic foragers. Was it possible for foragers to permanently occupy all desert areas or to inhabit them through all time periods^4,9,30^?

For more than 50 years anthropological and ethnoarchaeological studies have characterized the nature of economy and social life in historical desert societies as small, nomadic family groups of 10–30 individuals who foraged opportunistically across large territories of land, moving and targeting resources flexibly in response to resource availability^1^. Groups occasionally gathered in larger congregations, but for short periods and only during times of increased water abundance^1^. Archaeologists typically presume the past total human population in the Western Desert had remained comparatively low throughout time, but increasingly some argue that population increased substantially in the past 2000 years^17^. It was initially argued that low population density and opportunistic plant-based economic strategies are so essential in these deserts that they were always present in past behavioral systems, prompting the hypothesis that desert societies must have been culturally conservative and unable to change socially or economically over time^1^. However that conclusion is not supported by the serial changes observed in the archaeological record and models of desert conservatism are considered refuted^14,31,32^. A more plausible hypothesis is that past behavioral strategies varied in response to differing palaeoenvironmental conditions^7,13^. Evaluating that hypothesis requires observations of environmental as well as behavioral change over time and space.

The time span of human activity in the Western Desert region is staggering. The north and northwestern margins of the Western Desert were initially occupied by at least 45 ka^9,33^, and there was widespread occupation of all desert regions prior to ~ 35 ka^14,32^. At that time this region experienced a wetter climatic phase, with more reliable rainfall and lower evaporation and consequently surface water was in greater abundance than today^34–36^. Around 34 ka, a climatic shift towards the long, slow onset of the last glacial maximum (LGM) commenced, culminating in maximal glacial conditions between 24 and 18 ka^35^. This period was characterised by increased aridity and reduced predictability of rainfall, which in combination with more windy conditions, reduced vegetation cover and encouraged the development of extensive dunefields across the continental interior of Australia^36–38^. Deglacial/Early Holocene conditions beginning ~ 14 ka reversed this trend, with increased seasonal rainfall and increased vegetation cover^35,39^. The mid-Holocene period saw weakening of monsoonal activity and an extended phase of increased aridity, from approximately 5–3 ka^40,41^. The climate of the past 1500 years has been much like the present day^42^, and so can be effectively studied using modern environmental remote sensing data.

**Figure 2:**
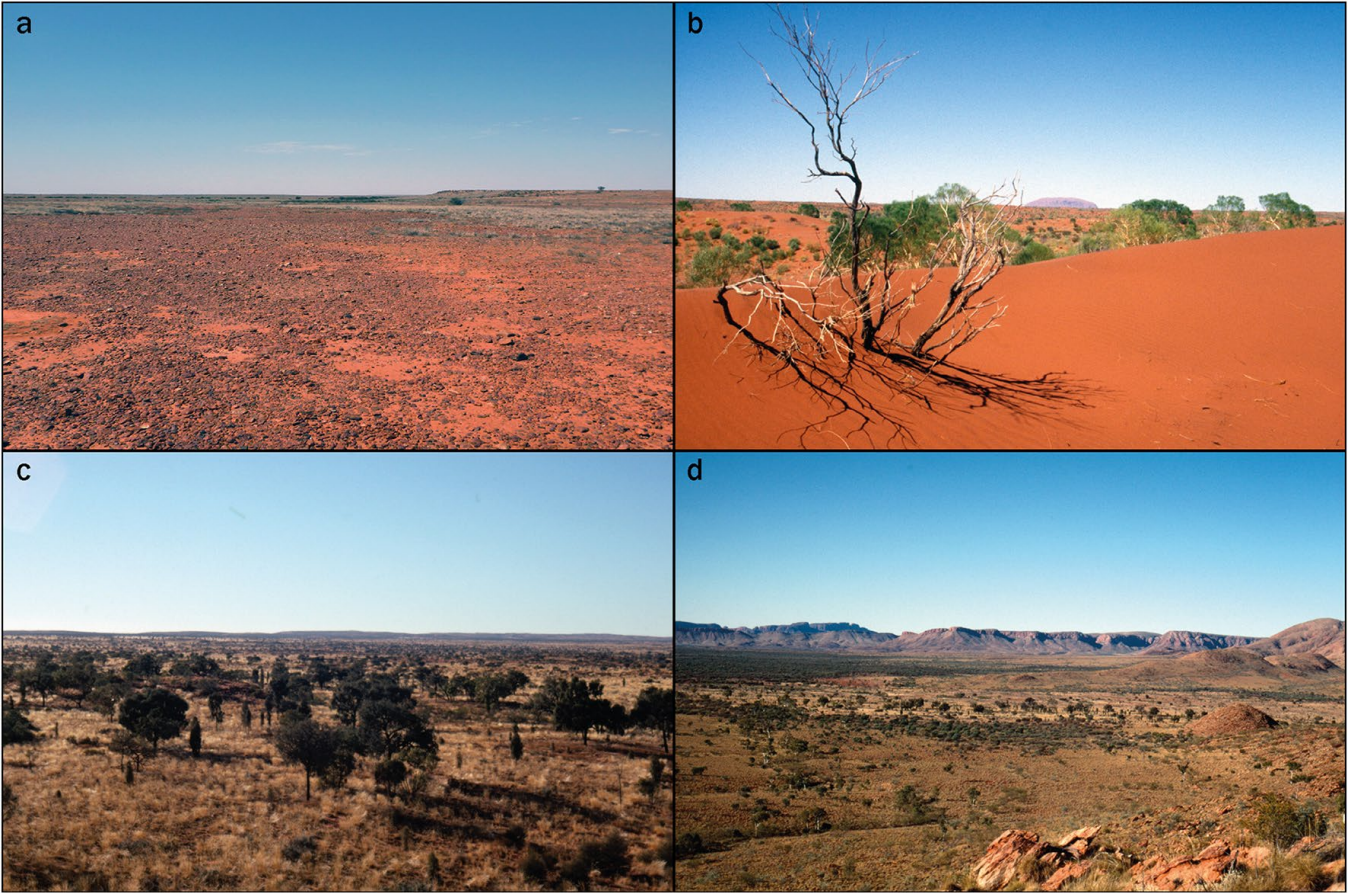
Photographic examples of coarse scale land systems of the Australian Western Desert. (**a**) Stony desert plains with scarce vegetation or substantial overstory. Vegetation is concentrated along drainages. (**b**) Sandridge desert with extensive linear dunefield. Dune ridges carry sparse perennial vegetation, while interdunal swales have intermittent, often dense ephemeral growth. (**c**) Sandplain lowland deserts with hummock grasslands (*Spinifex* spp.) and patchy *Acacia* spp. overstory. (**d**) Montane uplands and lower intra-upland areas. Intra-upland areas are more easily traversed and the vegetation patterning is similar to sandplains. Mountainous uplands, with deeply incised gullies and perennial resources, are argued to have functioned as refugia during extreme periods of aridity. Photos: W. Boone Law.

One of the major limitations in understanding variation in desert ecology and its implication for human land use has been the extremely coarse scale of environmental characterization used in archaeological and anthropological discussions of desert life. Archaeological models of LGM abandonment and post-LGM reoccupation exemplify the use of desert-scale units (Fig. 2). These models employed very broad regions as units of analysis, acting as though all sandridge deserts or all upland ranges were relatively similar and internally uniform in resources. For example, it has been argued that during the LGM, human populations largely abandoned lowland deserts while persisting in better resourced mountainous terrains that functioned as refugia^7,18^. And in the climatic amelioration following the LGM archaeologists debated whether all lowland systems were immediately reoccupied^5^ or whether only stony deserts and sandplains were utilized while sandridge deserts were unable to be used until c. 5000 years ago, when innovations in stone tool technologies and population changes encouraged expansion into dunefield land systems^4^. These coarse analytical scales provided researchers with a practical heuristic means with which to analyze and evaluate the dynamics of past land use^4^, and the research it generated demonstrated that some sandy lowland areas were occupied at times during the LGM and early Holocene^4,5,9,24,30,33^. These works illustrate how previous coarse scale approaches are now inappropriate because they fail to evaluate landscape use at the more localized scale of individual foragers and hide the variability present in the desert environment. Building on this proposition, we predict satellite imaging, environmental remote sensing, and geographic information systems offer the next step forward for sharpening our understanding human–environment interactions, as these technologies have the capacity to create finer-grained, human-scale depictions of Western Desert environments and a higher resolution image of foraging patch opportunity.

A number of assertions underlie standard discussions of Western Desert human ecology and its relationship to recent Aboriginal land use practices. Nearly all previous ecological models include the following propositions: (1) traditional forager subsistence and settlement was principally tethered to locations of water availability, closely followed by plant resources^1,2,43^, (2) past foragers prioritized their activities around ephemeral waters, and as those temporary resources depleted, foragers reorganized their land use around semi-permanent and permanent waters, (3) permanent waters were frequently common only in montane desert ranges which likely functioned as ecological refugia at times in the past^4,5^, and (4) variability amongst arid land systems and biogeographic areas presented distinctive economic challenges for resident populations, and the manner in which people adapted to and behaved in particular desert terrains are likely to be expressed in the archaeological record^4,5,7^. This last point, in which specific human economic behaviors are inextricably linked to particular but broad biogeographic regions, landforms, or environmental conditions^4^, has led to a coarse-grained understanding of Aboriginal ecology and the arid zone archaeological record, and in this article we argue that this proposition is problematic and should be abandoned.

All previous ecological models recognize the importance of water for successful arid settlement and subsistence. There is little argument that can be made against the importance of that resource. Without access to water, Aboriginal groups would not have been able to effectively forage and survive. Accordingly, some researchers emphasize the importance of the ‘connectedness’ of water resources across the arid zone, hypothesizing that water availability was the principal driver of human movement throughout desert regions^16^. However, human behavior is complex, and in all likelihood, additional environmental, geographical, technological, and demographic factors greatly influenced how land use and residential mobility was expressed^14^.

For example, water abundance was not only important for human life, but it was equally important for local flora and fauna, upon which past Aboriginal groups also relied. Most models therefore emphasise a strong link between water and vegetation^1,2,43^, reasoning that people would want to position themselves in way to maximize their plant foraging opportunities, not to mention hunt wild game attracted to the same resource.

It is also reasonable that differences in land systems and the general ruggedness of landscape would also affect land use and subsistence-settlement patterns. Human movement science has shown that terrain ruggedness is a prominent factor affecting movement trajectories, space use and energy costs^44,45^. Thus, terrain ruggedness is an important variable for understanding human behavioral ecology, in regards to energy expenditure and patch choice. Moreover, water availability and the distribution of plant communities varies between land systems due to soils, geology, and related terrain characteristics. Thus, as researchers before us, we suggest the variability of desert terrains affected past foraging behaviors, either knowingly or unconsciously, and influenced where groups situated themselves amongst water and plant resources.

Our research considers these earlier suppositions, which invariably influenced the structure and interpretation of our foraging suitability model. When modelled under the best known environmental conditions, we hypothesized that our ecological model would depict, in high spatial resolution, a wide range of suitable foraging habitats across the Western Desert and provide some indication of where desert dwellers could best position themselves amongst natural resources. We reason that this information would allow for us to critically evaluate the likely extent and permanency of Aboriginal occupation in the vast arid region and determine if land use was tied to particular land systems or bioregions, as suggested by others. Through the lens of this work, we suggest that our foraging suitability model holds implications for our understanding of precontact land use practices and the deep-time archaeological record itself.

## Results and discussion

**Figure 3:**
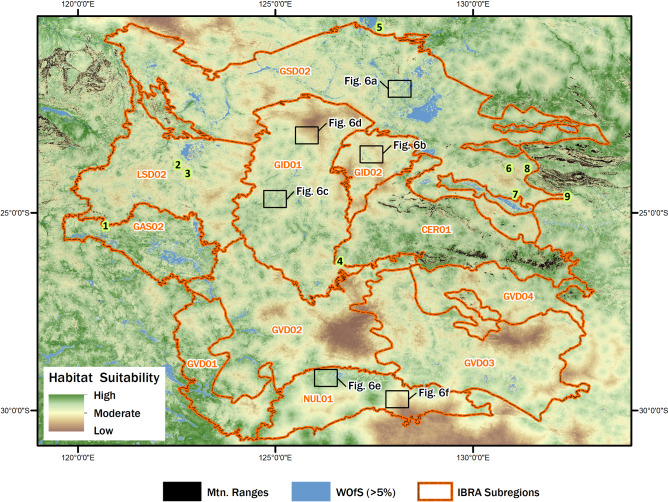
A satellite derived model of foraging habitat suitability for the Australian Western Desert. Foraging habitat suitability is highly variable within IBRA boundaries and throughout the Western Desert. Several massive areas of low-ranked foraging habitats are evident throughout the region. IBRA codes and excavated rockshelter sites (lime green- numbered) are defined in the Fig. 1 caption. Map created in ESRI ArcGIS Desktop 10.5.1 (https://desktop.arcgis.com), linear stretch (1.0%) visualization. See “Methods” section for source raster information.

‘Foraging habitat suitability’ is a reference to the favorability of a patch of land for day-to-day subsistence. Here, suitability is an index value ascribed to each potential foraging patch (grid cell) captured in a raster image, based on terrain movement costs and the proximity of each patch to water and green vegetation. We constructed our foraging habitat suitability model using satellite-derived environmental data, digital terrain information and anthropological field data on foraging range (Fig. 3). The model’s environmental foundation is based on more than two decades of continuous near bi-weekly Landsat-5 satellite observations, allowing for the systematic detection and measurement of water recurrence and vegetation condition for every 30-x-30 m image pixel. This period of observation is long enough to observe multiple fluctuations in this highly variable environment and to not be restricted to a single short-term climatic state, such as a bushfire or drought. Therefore the time frame provides a reliable observation and measurement of maximum vegetation greenness, regardless of temporary drops in NDVI. Similarly, maximum extent and occurrence of surface water is systematically measured through long-term satellite observations, avoiding measurements only of phases of drought or irregular rainfall. For this reason our model focuses on maximal values to represent the best environmental conditions that would have been available for past foraging activities since the last glacial, based on the contemporary climatic regime.

The model also uses the ALOS World 3D 30 m digital elevation data product to quantify terrain ruggedness across the study area^46^. Terrain ruggedness is a geomorphometric measure of land surface ruggedness, where elevation variability is used to infer ease of traversal when walking between locations in the landscape. Terrain ruggedness is suggestive of potential energy expenditure, assuming that increasingly rugged terrains necessitate higher levels of physical activity and caloric intake. Here, we integrated measures of ruggedness with environmental satellite data, providing an indication of which patches of vegetation and water are most easily accessed in regards to minimum changes in elevation.

Walking time to observed surface water is the final spatial parameter incorporated with the model. It is calculated using Tobler’s^47^ hiking algorithm and information on daily foraging practices. Historic anthropological data indicates Western Desert foraging activities typically operated for 4–6 h each day^1,48^, with foragers moving up to a day from ephemeral water sources in their food quest^1^. In accordance with these ethnographic statements we spatially delineated land areas where regular foraging activities may have occurred by first calculating the walking time from water, then weighting all areas that were less than 8 h walk from water more heavily in the input which went into our final suitability model. Since resources are said to be permanent in uplands^4,5^, we assume mountainous refugia were always suitable foraging habitats, so these refuge areas have been masked and removed from consideration (see mountain ranges in Fig. 3).

Appropriate elements from all of the aforementioned satellite datasets were combined to produce our foraging habitat suitability model (Fig. 3). The ~ 30 m spatial resolution of the data facilitates the construction of a spatially-explicit, geographically broad, yet fine-grained ecological model to visually observe and critically appraise foraging habitat suitability at a variety of scales, offering new perspectives on regional human behavioral ecology. The model provides a continuous ranking of the relative foraging value for each landscape patch (or 30 m grid cell in this instance). Interpretation of patch values is based on the proposition that foragers know the conditions in all parts of the landscape they visit, and they organized their daily foraging movements in accordance with the factors outlined above.

Our habitat suitability model illustrates the highly varied favorability of foraging patches across the Western Desert (Fig. 3), as calculated from data on natural resource distribution, terrain attributes, and daily foraging range. The model is conceptual, based on quantitative environmental variables that have been well documented to influence desert foraging activity. In regards to the model’s robustness, the input variables are equally weighted and statistically independent (see “Methods” section). The equal weighting reflects the concepts and assumptions of earlier research, particularly of existing landscape mapping, offering a coherent and consistent modelling approach. Advanced mathematical modelling, incorporating sensitivity analysis^49,50^, could be used to modify the weighted contribution of each variable, and such modelling will be the subject of future papers. Until more detailed knowledge of past forager land use and contemporary resources becomes available there is little benefit in arbitrarily substituting other input values in our model.

**Figure 4:**
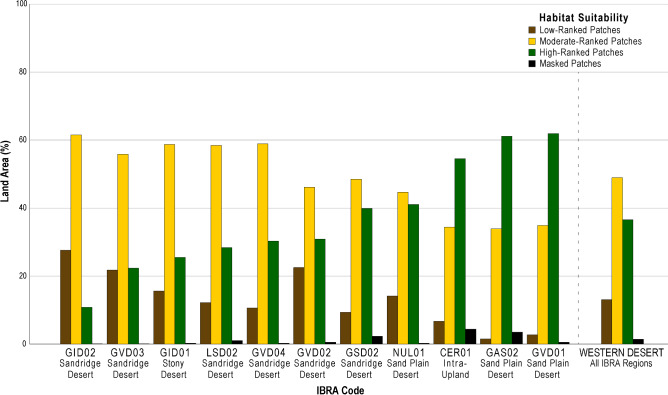
Percentage of land area (km^2^) occupied by low, moderate, and high-ranked habitat suitability patches for the eleven largest IBRA bioregions of the Western Desert (Table S1). The histogram is ordered left to right based of the percentage of high-ranked foraging habitat within each bioregion. The percentages for the entire Western Desert are presented on the far right.

The model comprises a matrix of nearly 1.3 billion data cells, each of which has been individually analyzed and ascribed each foraging patch a value indicative of potential habitat suitability. The computational power required to statistically analyze the dataset is massive, so to simplify computing and broadly characterize intraregional variation, we scaled up using nationally defined IBRA subregions. We used IBRA boundaries to group and rank the patch values into low, moderate, and high foraging habitat suitability classes and then calculated the land area occupied by each class (Fig. 4 and Table S1). Higher-ranked localities are well positioned in relation to suitable resources and easily traversed terrains. Lower-ranked patches are considered poorly-suited habitats due to their considerable distance from water and plant resources, and they are in comparatively rugged terrains. Areas deemed to have moderate foraging suitability have mixed accessibility to resources and variable terrain ruggedness.

The results show that during times of maximum water abundance and vegetation greenness, 36.6% of the Western Desert has high-ranked habitat suitability (Fig. 4 and Table S1). Moderately suitable areas constitute 48.9% and low-ranked patches encompass 13.1%. Breaking these findings down further, we calculated the ranked land areas for the eleven largest IBRA subregions (> 10,000 km^2^) of the Western Desert (Fig. 4 and Table S1). At a broad bioregional level, intra-upland zones (Fig. 4; CER01) and desert plains (Fig. 4; GAS02, GVD01, and NUL01) offer a greater percentage of high-ranked foraging habitats. Bioregions dominated by dunefields have considerably less high ranked land areas compared to uplands, plains, and areas of low relief, although it is important to note that there is also considerable patchiness amongst suitable foraging areas in sandridge desert regions (Fig. 3). For instance, the centrally located Gibson Desert dunefield area (Fig. 4; GID02) has very little area of high-ranked habitat (10.8%), which is far less than other sandridge desert bioregions (Fig. 4; GSD02, LSD02, GVD02, GVD03, and GVD04) where high-ranked suitability areas range between 22.3 and 39.9%. Similarly, the Gibson Desert stony desert bioregion (Fig. 4; GID01), which is dominated by lateritic surface gravels, records only 25.5% high-ranked habitat areas. Thus, at a coarse-grained scale, it seems that some central core regions of the Western Desert are more environmentally hostile and offer less high-ranked foraging opportunities compared to more peripheral bioregions. This generality does not imply such areas were unutilized by desert peoples, but rather some areas were on average volatile and had low productivity.

### Foraging potential is highly varied amongst bioregions and land systems

When viewed at a fine-grained scale, our model clearly shows that there is an uneven gradient of suitable foraging habitats across the Western Desert, and foraging suitability trends are not pervasive throughout particular bioregions or land systems (Fig. 3). Away from montane uplands, water permanence is always temporary, and land systems with low topography, such as plains, stony plains, and sandridge desert, have highly varied foraging suitability, even when characterized in the best environmental conditions.

The implications of this variation are important to understanding human ecology of the ethnohistoric period and the late Holocene archaeological record of the past 2000 years, when climatic conditions and landscapes were much like the present day^36,40,42^. Many scholars have noted that the historic desert peoples were familiar with the distribution of regional natural resources^1,5,7^. It has been argued that resource knowledge was articulated with socioeconomic strategies, and that groups routinely utilized all areas of the Western Desert during times of good rainfall and resource abundance. However, our suitability model reveals that there are large, expansive areas of the desert landscape that would have presented substantial challenges for survival, even in the best environmental circumstances (Fig. 3).

**Figure 5:**
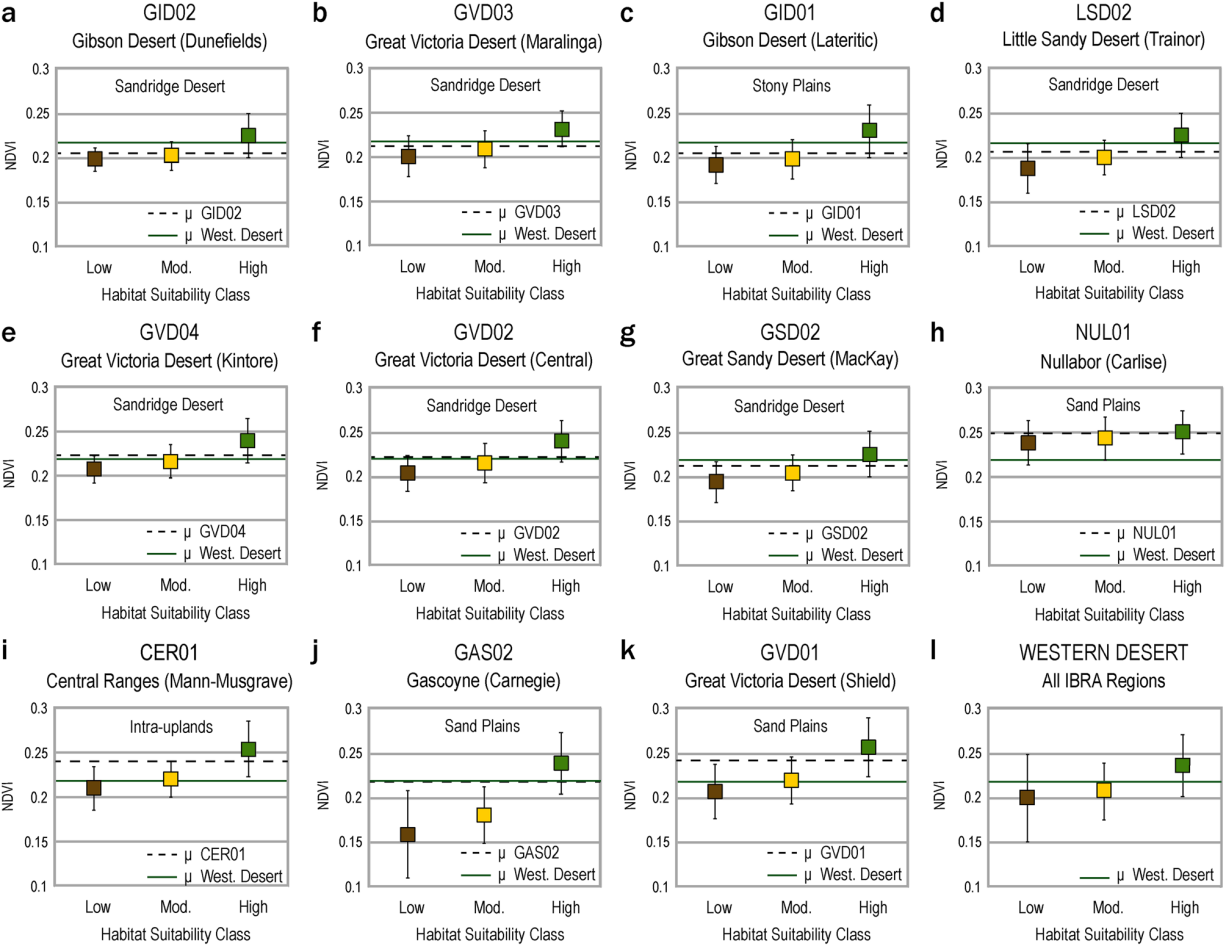
Boxplot of mean NDVI values and one standard deviation for low, moderate, and high-ranked suitability classes for the eleven largest IBRA subregions (**a**–**k**) of the Western Desert (**l**). Mean NDVI for individual bioregions and the spatial bounds of the Western Desert study area denoted as dashed black line and solid green line, respectively. IBRA subregion boxplot groups (**a**–**k**) are presented in order of increasing percentage of high-ranked foraging habitat, after Fig. 4. Table S2 offers the precise summarised NDVI values for each bioregion and suitability class.

Our model further suggests that low-ranked locations of foraging suitability were always below average productivity and were always comparatively unsuited as foraging habitats. To carry out that measure, we needed an independent indicator of land productivity, NDVI. We used satellite observations of maximum vegetation greenness to quantify how land productivity differs amongst low, moderate, and high ranked foraging habitats (Table S2). Variation in mean (µ) NDVI for each habitat class illustrates how land productivity differs within and amongst the most prominent Western Desert bioregions (Fig. 5). Given the below average NDVI of all low-ranked desert lowlands, we hypothesize that broad clusters of extremely unsuitable localities would be unlikely to provide adequate returns (Fig. 5), even when foragers were pursuing low-variance or lower quality resources^51^. Based on the distribution of low-ranked patches (Fig. 3), we agree with earlier research that the entire desert region was not equally economically viable for foraging, and that substantial tracts of land were not economically attractive to resident populations^4,5,14,32^. We also recognise that the distribution of massive-sized sub-optimal patches may be an important factor shaping the patterns of movement through the landscape, with foragers potentially preferring movement along high suitability corridors. However, unlike earlier research, our suitability model shows that unfavorable foraging areas are not correlated with large units of biogeography alone. Our model depicts the environmental variability of the Western Desert at a much higher resolution than its predecessors, revealing several massive land tracts where unfavorable foraging conditions occur (Fig. 3). If ethnographic patterns of land use were in place, we predict that many of these large areas would have been rarely utilized or perhaps some were purposefully avoided due to known deficiencies in the resource energy base^12^. This proposition is readily testable because it predicts that archaeological sites with poorly sorted, low densities of artefacts will be found in these places^12^. Defining the appropriate scale will be the key to testing our model, since we have demonstrated that broad biogeographic units are heterogenous and yet at a fine-grained scale, small areas of low suitability, which are often a local geographic feature (e.g., sand dune, bare rock outcrop, or erosional area), need not have been obstacles. Model testing will need an intermediate scale commensurate with daily foraging radii.

At present, the archaeological land use pattern of low-ranked foraging habitats is not something that is well-understood from the Western Desert, although periodic and short term use of impoverished, low productivity patches has been predicted^12^. Studies of contemporary Western Desert groups indicate that human-induced firing of the landscape enhances biodiversity and land productivity^51–54^, so it is possible that low productivity patches may have occasionally benefited from anthropogenic burning, especially in the past 1500 years^51^. However, research also suggests that cultural burning practices did not have widespread regional impacts^51–55^. Human influence on landscape modification is localized within day-range foraging areas around residential camps and frequently traversed pathways^51–54^. Low productivity patches away from residential camps were probably unlikely targets for either anthropogenic burning or foraging if higher-ranked patches were closer.

Elsewhere, in the eastern Australian arid zone, periodic use of climatically harsh desert localities is known from archaeological sequences. While in some cases preservation may explain chronological discontinuities^56^, there is compelling evidence for irregular occupation in several desert areas^10,57–60^. For instance, in the western Strzelecki Desert broad portions of dunefield landscapes were periodically abandoned for centuries or even millennia^57,60^, while in semi-arid portions of southeastern Australia sequences of occupation were separated by decades or centuries of local/regional abandonment^58,59^. Fluctuations in local foraging suitability may well be a factor producing discontinuous land use across the Australian arid lands, and we suggest that in the Western Desert there were patches with chronologically varying foraging potential. The key test of this prediction would be to investigate whether archaeological sites in locations of fluctuating habitat suitability over time also display histories of discontinuous visitation. Such sites could be identified through local palaeoenvironmental records but we suggest that selections based on time-series analysis of vegetation greenness from the past few decades would be more readily used to establish samples and would facilitate comparison of archaeological sites in terms of local foraging suitability and NDVI values, as well as archaeological records of continuous or discontinuous visitation.

### Satellite data reveals a more nuanced understanding of land use

Australian archaeological research has relied heavily on biogeographic principles to distinguish the ‘barriers and boundaries’ of Aboriginal subsistence and settlement in the arid zone^4,5,61^. While equating particular land use practices with specific bioregional areas was initially useful for generalized conceptualizations of traditional foraging behaviors, the coarse analytical scale of earlier approaches is now problematic. Subsequent research has shown the dynamics of Aboriginal occupation and land usage in the Western Desert to be more complex and variable across spatial and temporal scales than originally conceived^9,24,30,33^. To gain a more nuanced understanding of past land use and foraging patterns, finer-scale methods of analysis are required.

**Figure 6:**
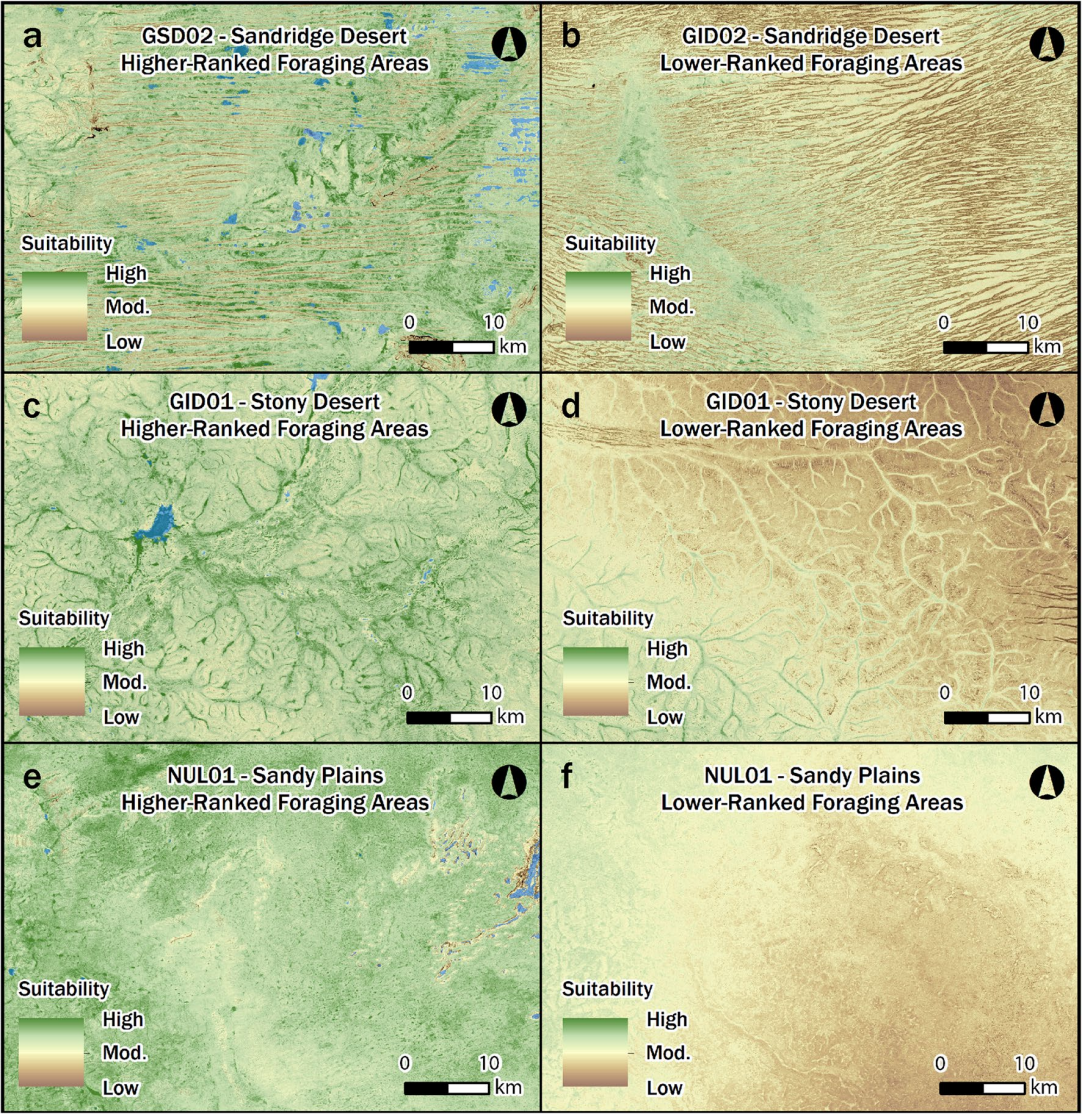
High resolution perspectives of various Western Desert landforms (e.g., sandridge, stony plain and sandy plain contexts) with generally higher-ranked and lower-ranked areas of foraging suitability. This figure illustrates the fine-grained scale of our habitat suitability model (Fig. 3), which has implications for better understanding localized land use behaviors. Juxtaposed areas, as mapped in Fig. 3 are: (**a**) Higher-ranked sandridge habitats vs. (**b**) lower-ranked sandridge land system. (**c**) Higher-ranked stony desert habitat vs. (**d**) lower-ranked stony desert areas. (**e**) Higher-ranked sandplain land systems vs. (**f**) lower-ranked plain habitats. Maps created in ESRI ArcGIS Desktop 10.5.1 (https://desktop.arcgis.com), linear stretch (1.0%) visualization. See “Methods” section for source raster information.

We used satellite imagery to tackle the issue of scale, allowing for a sharper and more spatially explicit examination of desert environments and landscapes. For example, as we focus at higher resolution on various areas of the Western Desert, our model clearly shows that foraging suitability is highly varied across all desert lowlands (Figs. 3, 4 and 6). In sandridge desert areas, proposed to have been a barrier at times in the past^4^, the model shows there are many well-watered and amply vegetated localities where good foraging is possible when rainfall is high and surface water is abundant (Fig. 6a). In this context, interdunal swales are hardly barriers to occupation because they can be lush with water, plant, and wildlife resources after local rain, and the energy expenditure required to walk along interdunal swales is low in comparison to the requirements needed to repeatedly scramble across a sea of loose sands and undulating dunes. Thus, it seems entirely plausible that resident groups could navigate and forage in many dunefield areas by following a well-resourced network of swales during times of good environmental conditions. The fine-grained nature of this observation opens up the possibility that many sandridge deserts were not necessarily broad barriers to occupation and that precontact land use behaviors varied in different dunefield contexts.

We also highlight that the resource-rich swale pattern is not found in all dune systems (Fig. 6b), and it is plausible that some of these areas were periodic barriers to occupation, as previously suggested in more generalized ecological models^4^. There are substantial areas of sandridge desert, especially within central areas of the Western Desert (e.g., GID02), where survival would have always been extremely difficult, even during times of abundance (Fig. 6b). This variability is also expressed in stony desert contexts, where southern areas of the lateritic Gibson Desert (GID01) offer better habitat suitability (Fig. 6c) than the northern areas (Fig. 6d). On a fine scale, plain land systems also exhibit a wide range of habitat suitability, where high-ranked habitat suitability appears fairly widespread in some areas of the Nullabor Plain (NUL01; Fig. 6e), yet other areas of the plain were poorly-suited for foraging (Fig. 6f).

In previous ecological models, stony desert and plain land systems are considered more favorable than sandridge desert^4^; however, as shown above, the modelled data clearly illustrate that there are substantial areas of plains and stony desert landscapes that vary considerably between high and low-ranked habitat suitability (Figs. 3, 4 and 6). The fine-grained scale of our model adds to a growing body of research^5,9,24,30,33^ that demonstrates how previous pan-continental characterizations of deserts as ‘corridors’ and ‘barriers’ for foragers oversimplify the link between human behavior and biogeography. When scrutinized at high resolution, extremely unsuitable foraging and very well-suited foraging areas can potentially occur in any area of the Western Desert, regardless of the biogeography or other physical characteristics. Thus, fine-grained ecological models allow for a more nuanced and spatially-explicit understanding of the past land use behaviors that led to the formation the desert archaeological record.

### Using environmental remote sensing to infer LGM habitat suitability

There is no doubt that the Western Desert environment has changed and evolved over time, through both natural and human-induced processes^8,36,37,51^. The region has undergone considerable environmental fluctuations over time, resulting in landform transformations (dune aggradation, in particular) and changes in vegetation cover. The long-term physical impact of these environmental changes clearly place limitations on how modern satellite data can be used to interpret deep-time patterns of occupation and land use. However, our model shows the likely distribution of low-ranked foraging habitats when climatic conditions were much drier than present.

Ethnoarchaeological accounts depict resident populations as being low density and highly mobile, frequently moving and foraging across vast expanses of territory, thereby necessitating intermittent patterns of settlement^1–4^. Such a mobile strategy means that large swathes of the desert could not have been continuously occupied. Our habitat suitability model (Fig. 3) makes sense of the impermanent and mobile land use strategy seen historically. For example, we document several massive areas of the Western Desert where, in combination, surface terrains are physically challenging, the nearest proximity to surface water is greater than 2 days walk, and vegetation cover, density and condition is substandard, even in the best of documented environmental conditions! These exceptionally large areas were poorly-suited to foraging, and could not have been permanently occupied in the historical period. They would only have been visited rarely, perhaps only in atypical short-term climatic events, and may even have constrained forger movement between more favorable parts of their territories. Given current palaeoclimatic evidence we infer that in the Pleistocene these low ranked habitats would have been even more inhospitable to foragers than in recent times. During the LGM, the resource yields in such areas would have been more diminished than present, making conditions for survival even more difficult than today. Consequently, we predict that unless radically different economic strategies were being employed in the Pleistocene those areas would have been only rarely visited since the peak of the last glacial cycle, ~ 24–18 ka, even though adjacent desert areas may have supported regular or at least sporadic visitation.

Our hypothesis is clear, detailed, and framed to be testable by archaeological fieldwork. The number of Western Desert sites with old archaeological sequences is growing, but the sample is small, site distribution is widely scattered, and none are located in the harsher core areas identified in this study (Figs. 1 and 3). Thus, it is evident that archaeological fieldwork in those impoverished landscapes as well as environmentally richer and more reliable landscapes is necessary to understand historical land use patterns and to make statements about earlier phases of regional occupation. Our work highlights how future models of forager land use across Australia’s desert regions can comprehend the environmental complexity and fine scale of resource variability in these vast, remote and diverse places.

## Methods

### Software and data sources

ArcGIS Desktop 10.5.1(ArcMap) with the Spatial Analyst Extension was the principal software package used for analysis and spatial modeling^62^, with additional software used for some pre-processing tasks. The Landsat NDVI time series was accessed via Google Earth Engine (GEE), a cloud-based geospatial platform^63^. QGIS 2.4, also an open-platform GIS package, was used for terrain ruggedness processing^64^.

All of the Earth observation datasets described below offer continental-wide spatial coverage and are available as georeferenced ~ 30 m spatial resolution packages. For ease of processing, our area of interest was clipped and mosaicked as appropriate, and raster cell grids were aligned using the snap raster function in ArcMap 10.5.

Water Observations from Space (WOfS) is a Geoscience Australia time-series dataset developed from imagery acquired by NASA’s Landsat 5 and Landsat 7 satellites from 1987 to 2014^19^. The summary dataset product, used here, was produced from more than 184,500 scenes collected over this 27 year period^65^. The summary product is a single band raster depicting the recurrence of detectable surface water in an area of land. The water observations are recorded as a percentage for each grid cell, which Geoscience Australia calculated by using the ratio of detected surface water observations to the number of clear observations for all acceptable Landsat scenes.

The Landsat 5 TM 8-Day NDVI composite image collection is a time-series datacube or pre-processed NDVI scenes collected between 1984 and 2012^20^. The Landsat 5 TM 8-Day NDVI collection was produced by the United States Geological Survey using top-of-atmosphere reflectance to calculate NDVI globally at 30 m spatial resolution^66^. The collection is stored in Google Cloud, where it can be freely accessed and processed via GEE^63^. NDVI is a widely used remote sensing index that summarizes vegetation greenness. It is a measured inference of plant cover, biomass and condition, based on how light is reflected in the near-infrared (NIR) and red (Red) spectrum of the Landsat 5 imagery. NDVI was calculated using the ratio: (NIR − Red)/(NIR + Red). NDVI values range − 1.0 to 1.0, where higher values indicate more actively growing or extensive vegetation and values near or slightly above 0.0 suggest bare earth.

The ALOS World 3D 30 m grid digital surface model (DSM) product is available free of charge from Japan Aerospace Exploration Agency. The product is a 30 m resolution continuous topographic model of the Earth with ± 5 m vertical height accuracy^67,68^.

### Derived variable 1 (v*ar*1): water accessibility

WOfS time-series summary raster data was visualized in ArcMap and filtered to display all localities where surface water has been detected in greater than 5.0% of satellite observations. These localities represent the maximum abundance of surface water. For classificatory purposes, the water bodies were converted to a binary raster showing detected water locations (n = 1) and cells with no detectable water (n = 0). Walking time to detected water localities was calculated using the ArcMap’s path distance tool, using the ALOS World 3D 30 m and Tobler’s hiking function as vertical factor parameters^47^. The end-product is a single-band continuous raster with cell values representing the walking time (hours) to the nearest detected source of water. Raster values ranged from 0.0 to 36.5 h walking distance to nearest water. ArcMap’s fuzzy linear membership function was used to reclassify and transform walking data to a 0 to 1 scale based on criteria supported by ethnographic foraging observations^1,48^. Localities between 0 to 16 h walking time were reclassified along a linear scale of partial membership, where land areas immediately adjacent to waterhole were assigned a full membership value of 1, areas 8 h distant were assigned a partial membership value of 0.5, and areas greater than 16 h walking distance were classified with a membership value of 0.0, indicating they are undesirable due their distant proximity to water (Fig. S1).

### Derived variable 2 (var2): maximum vegetation greenness

Landsat 5 TM 8-Day NDVI composite image collection (1 January 1984–8 May 2012) was analyzed using the Google Earth Engine code editor^63^. The image collection was processed with the ‘reducer.percentile’ function code to produce a single band raster showing 95th percentile NDVI values. This composite NDVI image represents the maximum vegetation greenness values observed over more than two decades. The 95th percentile NDVI image was exported as a georeferenced .TIF file and the ArcMap 10.5 fuzzy linear function used to reclassify and transformed the NDVI values to a 0 to 1 scale. NDVI values greater than or equal to 0.3 were assigned a membership value 1, suggesting healthy vegetation (note: the mean NDVI of healthy grass in arid Australia is 0.29^69^). Assignment of fuzzy membership values was decreased along a linear scale between NDVI values of 0.3 and 0.0, where, for example, an NDVI value of 0.15 was assigned a partial membership value of 0.5 and NDVI values equal to or below 0.0 were assigned a membership value 0, indicating bare earth (Fig. S2).

### Derived variable 3 (var3): terrain ruggedness

The terrain ruggedness index (TRI) is a measure of topographic variability, calculated as the mean difference between the elevation of a central pixel and its surrounding cell elevations. The ALOS World 3D 30 m DSM was used to calculate the TRI raster in accordance with previously published conventions^46^. The TRI .TIF raster was created in QGIS using the terrain analysis tool package, and TRI cell values were visually analyzed to determine break points for landform patterning. In the context of the Western Desert, TRI values less than 3.5 equate to relatively level land surfaces, values 3.5–17 demarcate sand dunes and basal scree slopes, and values greater than 17 delineate escarpments and upland areas. A fuzzy linear function was applied to reclassify and transform the TRI data to a 0 to 1 scale. A TRI value of 0 is considered to have a membership value of 1, indicating easily traversed, flat terrain. Fuzzy membership values scaled downward between TRI values 1 and 17, where, for instance, a TRI value of 8.5 was assigned partial membership of 0.5. All TRI values greater than or equal to 17 were assigned a membership value of 0, indicating rough upland terrains, which correspond well with refugia areas (Fig. S3).

### Modelling foraging habitat suitability and classifying suitability areas

Prior to modelling, the statistical independence of the three derived input variables (var1, var2, and var3) were established using the correlation matrix function in the ‘band collection statistics’ tool in ArcMap (var1:var2, − 0.05404; var1:var3, − 0.04500; var2:var3, 0.12185). The foraging habitat suitability model, S, was built using the ArcMap raster calculator and the equation:

$$ S = \frac{{\left( {var1 + var2 + var3} \right)}}{N} $$

where var1, var2, and var3 correspond to the derived variables described above and N is the total number of input variables, which in this instance is 3. The raster calculator produced our foraging suitability model as a single band continuous. TIF image, with values between 0 and 1. The result is a negative skewed distribution of values, where the mean is 0.803.

For analytical purposes, the Jenks natural breaks method was used to statistically cluster and reclassify the grid cell values into a raster depicting three habitat suitability classes (Fig. S4). The Jenks method is sometimes called the ‘goodness of variance fit’, because it minimizes the average deviation from the mean of each group and maximizes the deviation from the means of other groups, thereby identifying natural classes amongst the data range. Data values greater that 0.829 were classified as high-ranked habitat suitability. Values between 0.710 and 0.829 were ranked have moderately-ranked habitat suitability. Values lower than 0.710 represent areas with low-ranked habitat suitability.

### Zonal statistics

ArcMap’s ‘zonal statistics as table’ function was used to extract land area statistics and maximum greenness values from the 95th percentile NDVI values (var2). The function uses a defined raster zone (e.g., IBRA region or habitat suitability class) to extract and provide summary calculations for cell values within a zone, which is defined as an input area where a group of cells have the same value. In this instance, IBRA subregions (17 classes) and habitat suitability classes (low, moderate, and high-ranked; Fig. S4) were used as zones to extract NDVI values and calculate the number of pixels, spatial area (km^2^), mean, minimum, maximum, and standard deviation of cell values for each respective zone. The results of these extracted values are presented in Tables S1 and S2. Unless indicated otherwise, maximum surface water observations (var1 values > 5) and montane uplands (var3 values > 17) were masked from analysis.

## Supplementary Information

Supplementary Information.

## Data Availability

The Interim Biogeographic Regionalisation for Australia (Version 7) and the Water Observations from Space data products are available at the Australian open government data portal https://data.gov.au. The Landsat 5 TM Collection 1 Tier 1 8-Day NDVI composite can be found in the Google Earth Engine Data Catalog https://developers.google.com/earth-engine/datasets. The ALOS World 3D 30 m (AW3D30) digital surface model data is available from the Japan Aerospace Exploration Agency Earth Observation Research Center https://www.eorc.jaxa.jp/ALOS/en/aw3d30/data/index.htm. All information needed to reduce the data and evaluate the conclusions is presented in the published methods. The Western Desert foraging habitat suitability model is available for use from the corresponding author, subject to a collaborative research agreement and licensing conditions.
