# Supplementary material for: Using satellite imagery to evaluate precontact Aboriginal foraging habitats in the Australian Western Desert
Source: Sci Rep. 2021 May 25;11:10755. doi: 10.1038/s41598-021-89642-1 (PMC8149716; doi:10.1038/s41598-021-89642-1)
Supplement: Supplementary file 1 — Supplementary Information. [file 41598_2021_89642_MOESM1_ESM.pdf]

Title: Using satellite imagery to evaluate precontact Aboriginal foraging habitats in the Australian Western Desert.

Authors: W. Boone Law*, Peter Hiscock, Bertram Ostendorf, and Megan Lewis

*Correspondence to: wallace.law@adelaide.edu.au.

SUPPLEMENTARY MATERIALS

Table S1

Land area statistics for Western Desert IBRA subregions, presented by foraging habitat suitability class.

				Habitat Suitability Class							
				Low-Ranked		Moderate-Ranked		High-Ranked		Masked Area	
Code	IBRA Name and Landform Type	Pixel Count	Area km²	km²	%	km²	%	km²	%	km²	%
CER01	Central Ranges (Mann-Musgrave) Intra-uplands	108491446	92997.85	6201.00	6.7%	31956.44	34.4%	50721.68	54.5%	4118.73	4.4%
CER02	Central Ranges (Watarru) Intra-uplands	4994548	4281.28	585.14	13.7%	2769.53	64.7%	860.18	20.1%	66.43	1.6%
GAS02	Gascoyne (Carnegie) Sand Plain	55032835	47173.63	723.29	1.5%	15995.76	33.9%	28809.74	61.1%	1644.83	3.5%
GID01	Gibson Desert (Lateritic) Stony Plain	148492069	127286.01	19913.67	15.6%	74658.07	58.7%	32444.31	25.5%	269.97	0.2%
GID02	Gibson Desert (Dunefield) Sandridge	34144136	29268.03	8070.02	27.6%	17995.97	61.5%	3175.52	10.8%	26.52	0.1%
GSD02	Great Sandy Desert (Mackay) Sandridge	301656747	258577.35	24118.59	9.3%	125407.23	48.5%	103124.21	39.9%	5927.31	2.3%
GSD03	Great Sandy Desert (Ehernberg) Intra-uplands	4445630	3810.75	67.08	1.8%	968.11	25.4%	2706.08	71.0%	69.48	1.8%
GSD04	Great Sandy Desert (Amadeus) Salt Lake	8542556	7322.60	295.08	4.0%	2567.29	35.1%	3897.29	53.2%	562.93	7.7%
GSD05	Great Sandy Desert (Lake Bennett) Salt Lake	3440296	2948.99	44.09	1.5%	191.95	6.5%	2578.89	87.5%	134.06	4.5%
GSD06	Great Sandy Desert (Lake Lewis) Salt Lake	989227	847.96	26.87	3.2%	199.59	23.5%	513.17	60.5%	108.33	12.8%
GVD01	Great Victoria Desert (Shield) Sand Plain	55283579	47388.57	1276.92	2.7%	16551.90	34.9%	29321.03	61.9%	238.72	0.5%
GVD02	Great Victoria Desert (Central) Sandridge	147028966	126031.86	28377.87	22.5%	58045.83	46.1%	38916.94	30.9%	691.21	0.5%
GVD03	Great Victoria Desert (Maralinga) Sandridge	135215812	115905.73	25291.10	21.8%	64625.07	55.8%	25817.48	22.3%	172.08	0.1%
GVD04	Great Victoria Desert (Kintore) Sandridge	58646558	50271.28	5308.36	10.6%	29620.91	58.9%	15248.84	30.3%	93.17	0.2%
LSD01	Little Sandy Desert (Rudall) Intra-uplands	11557071	9906.61	859.95	8.7%	3163.76	31.9%	5534.43	55.9%	348.48	3.5%
LSD02	Little Sandy Desert (Trainor) Sandridge	117770723	100951.96	12267.74	12.2%	58968.00	58.4%	28686.56	28.4%	1029.66	1.0%
NUL01	Nullarbor (Carlisle) Sand Plain	67764672	58087.24	8193.75	14.1%	25923.24	44.6%	23879.57	41.1%	90.68	0.2%
Western Desert	All Subregions	1263496871	1083057.72	141620.53	13.1%	529608.66	48.9%	396235.93	36.6%	15592.60	1.4%

Table S2

NDVI statistics for the eleven largest Western Desert IBRA subregions (>10,000 km^2), presented by habitat suitability class (see Fig. 5).

IBRA Code	Suitability Class	Pixel Count	Area km^2	Min.	Max.	Range	Mean	St.Dev.
GID01	Low	23231318	19913.67	0.041	0.450	0.409	0.192	0.021
	Moderate	87096221	74658.07	0.043	0.516	0.473	0.198	0.023
	High	37849582	32444.31	0.147	0.568	0.422	0.230	0.030
	All GID01*	148177121	127016.04	0.041	0.568	0.527	0.205	0.029
GID02	Low	9414504	8070.02	0.077	0.396	0.319	0.198	0.013
	Moderate	20994128	17995.97	0.079	0.464	0.385	0.202	0.016
	High	3704566	3175.52	0.147	0.473	0.325	0.225	0.025
	All GID02*	34113198	29241.51	0.077	0.473	0.396	0.203	0.018
GSD02	Low	28136787	24118.59	0.023	0.588	0.565	0.194	0.023
	Moderate	146300280	125407.23	0.039	0.673	0.634	0.203	0.020
	High	120304868	103124.21	0.146	0.763	0.617	0.224	0.025
	All GSD02*	294741935	252650.03	0.023	0.763	0.740	0.211	0.026
LSD02	Low	14311563	12267.74	0.029	0.602	0.573	0.187	0.028
	Moderate	68792161	58968.00	0.039	0.583	0.544	0.199	0.019
	High	33465792	28686.56	0.147	0.589	0.442	0.224	0.025
	All LSD02*	116569516	99922.30	0.029	0.602	0.573	0.205	0.026
GAS02	Low	843797	723.29	0.040	0.607	0.568	0.158	0.049
	Moderate	18660685	15995.76	0.052	0.607	0.556	0.180	0.032
	High	33609493	28809.74	0.147	0.595	0.449	0.238	0.035
	All GAS02*	53113975	45528.80	0.040	0.607	0.568	0.216	0.045
GVD01	Low	1489656	1276.92	0.057	0.404	0.347	0.206	0.030
	Moderate	19309475	16551.90	0.059	0.527	0.468	0.219	0.026
	High	34205956	29321.03	0.147	0.559	0.412	0.256	0.034
	All GVD01*	55005087	47149.85	0.057	0.559	0.502	0.241	0.036
GVD02	Low	33105672	28377.87	0.051	0.454	0.403	0.204	0.020
	Moderate	67716362	58045.83	0.055	0.486	0.431	0.215	0.022
	High	45400560	38916.94	0.147	0.594	0.448	0.239	0.024
	All GVD02*	146222594	125340.64	0.051	0.594	0.543	0.220	0.026
GVD03	Low	29504638	25291.10	0.034	0.396	0.361	0.200	0.023
	Moderate	75391711	64625.07	0.041	0.436	0.395	0.209	0.020
	High	30118717	25817.48	0.147	0.516	0.369	0.230	0.020
	All GVD03*	135015066	115733.65	0.034	0.516	0.481	0.212	0.024
GVD04	Low	6192746	5308.36	0.066	0.630	0.564	0.206	0.016
	Moderate	34555803	29620.91	0.070	0.670	0.600	0.215	0.019
	High	17789317	15248.84	0.148	0.568	0.420	0.239	0.025
	All GVD04*	58537866	50178.11	0.066	0.670	0.604	0.222	0.024
NUL01	Low	9558842	8193.75	0.061	0.546	0.485	0.238	0.024
	Moderate	30242094	25923.24	0.063	0.557	0.494	0.243	0.024
	High	27857950	23879.57	0.147	0.609	0.462	0.250	0.025
	All NUL01*	67658886	57996.57	0.061	0.609	0.548	0.245	0.025
CER01	Low	7234098	6201.00	0.070	0.533	0.464	0.210	0.024
	Moderate	37280435	31956.44	0.071	0.595	0.524	0.219	0.020
	High	59171999	50721.68	0.147	0.662	0.515	0.253	0.031
	All CER01*	103686532	88879.13	0.070	0.662	0.592	0.238	0.032
Western Desert	Low	165214734	141620.53	0.023	0.630	0.607	0.200	0.026
(All Subregions)	Moderate	617842315	529608.66	0.039	0.673	0.634	0.207	0.025
	High	462249472	396235.93	0.146	0.763	0.617	0.236	0.030
	All Subregions*	1245306521	1067465.12	0.023	0.763	0.740	0.217	0.031

*Masked pixels omitted from calculations

Figure S1

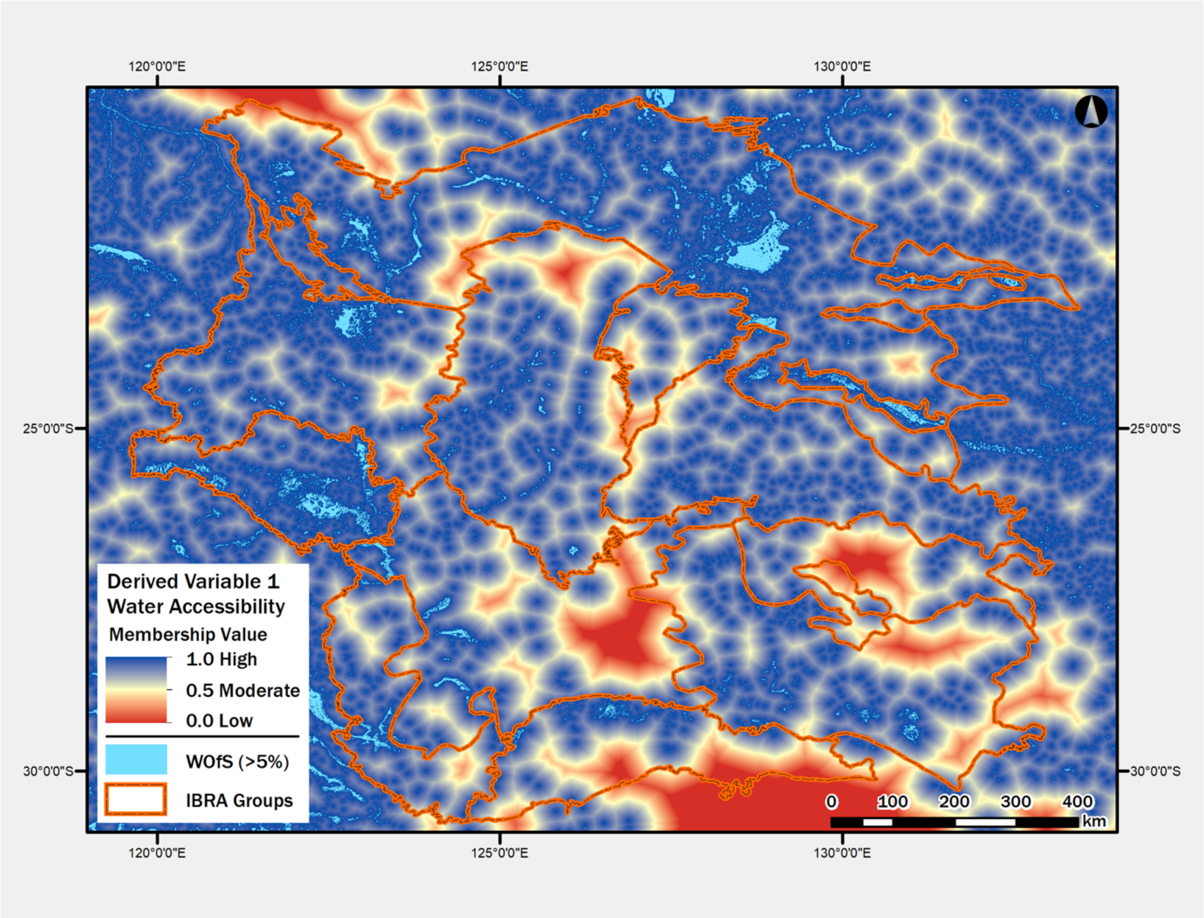

Fig. S1. Derived Variable 1 (*var*1) – Water Accessibility raster data image used as a foraging habitat suitability model input (see Methods). The image depicts fuzzy membership values based on proximity (walking time) to WOfS surface waters observed by Landsat-5 from 1987 to 2014. Map created in ArcGIS Desktop 10.5.1 (https://desktop.arcgis.com), linear stretch (1.0%) visualization.

Figure S2

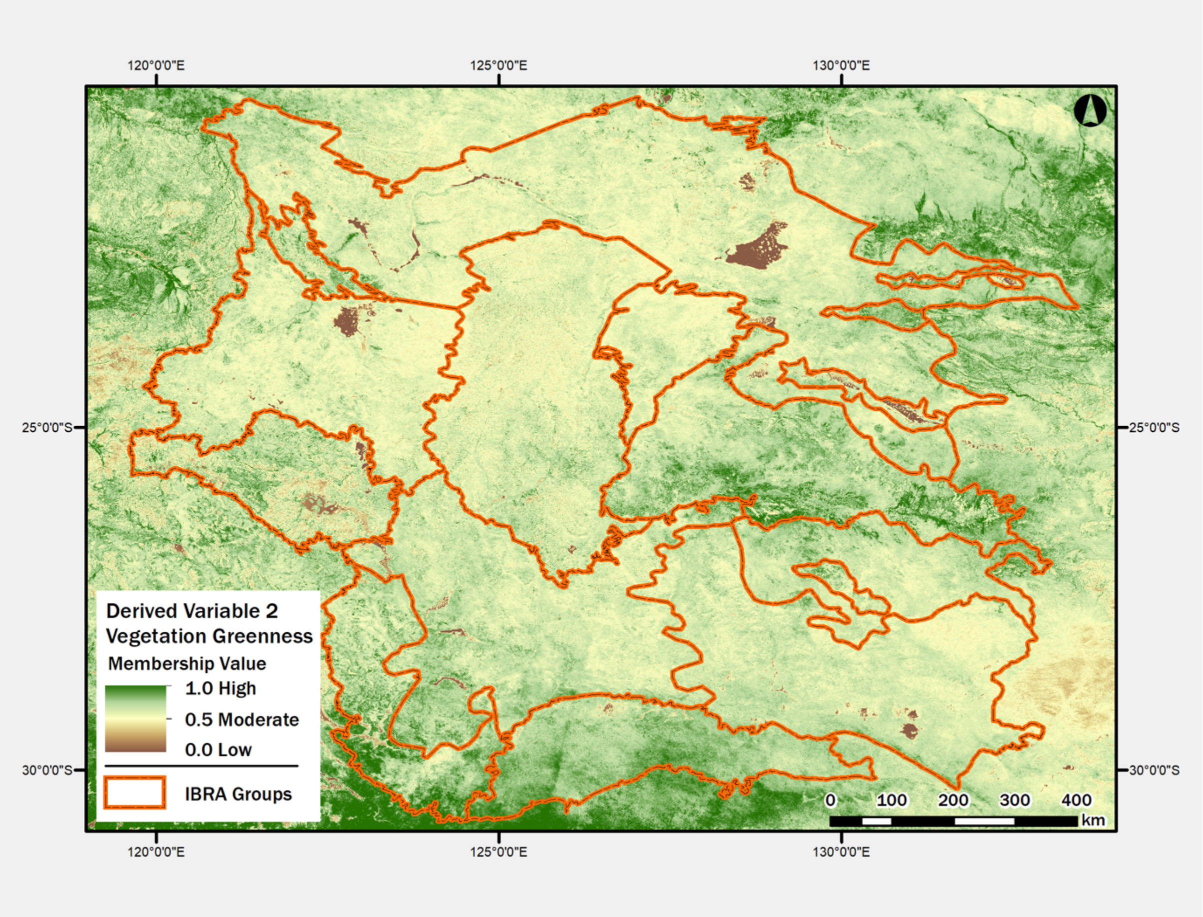

Fig. S2. Derived Variable 2 (*var*2) – Maximum vegetation greenness raster data image used as a foraging habitat suitability model input (see Methods). The image depicts fuzzy membership values based on maximum NDVI (95th percentile) values observed in near bi-weekly satellite observations from by Landsat-5 from 1984 to 2012. Map created in ArcGIS Desktop 10.5.1 (https://desktop.arcgis.com), linear stretch (1.0%) visualization.

Figure S3

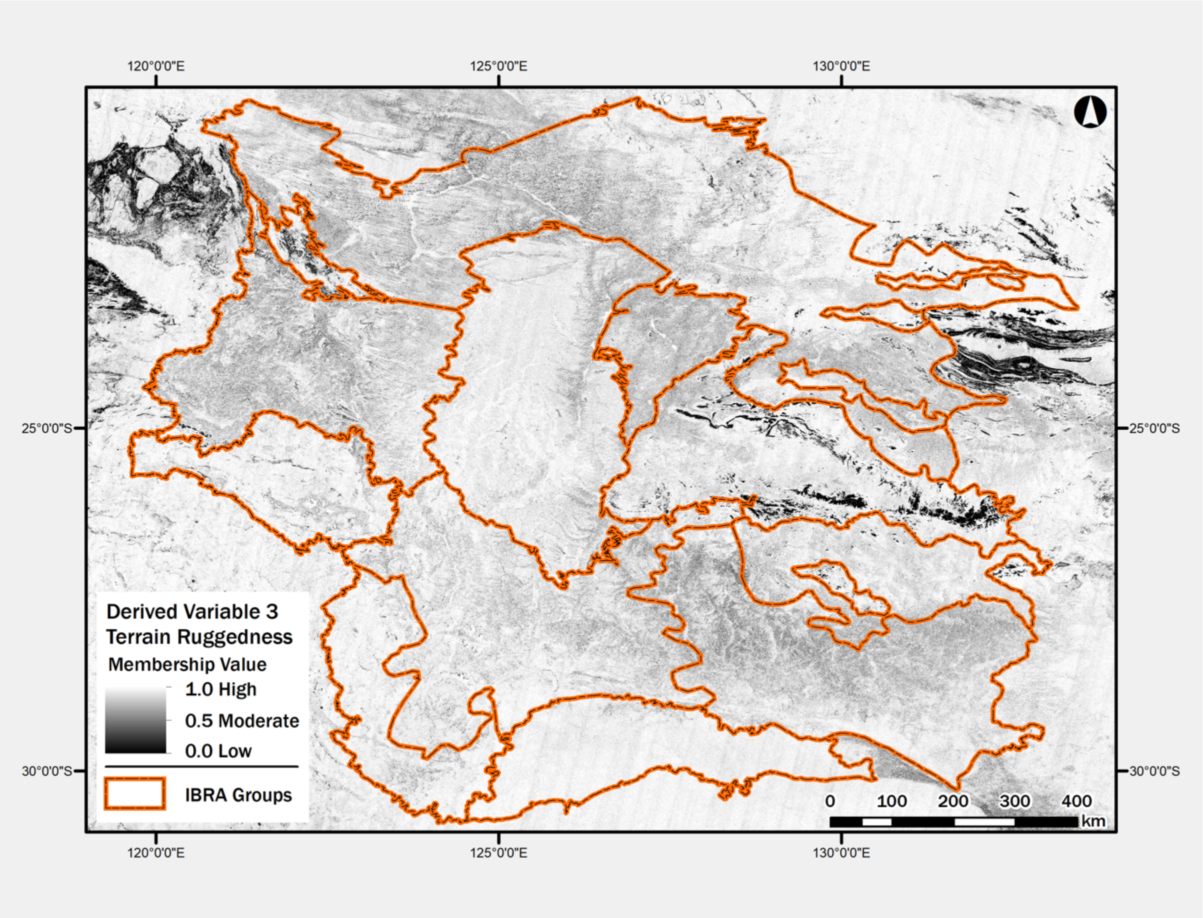

Fig. S3. Derived Variable 3 (v*ar*3) – Terrain Ruggedness raster data image used as a foraging habitat suitability model input (see Methods). The image depicts fuzzy membership values based on terrain ruggedness index model derived from the ALOS World 3D 30 m digital surface model. Map created in ArcGIS Desktop 10.5.1 (https://desktop.arcgis.com), linear stretch (1.0%) visualization.

Figure S4

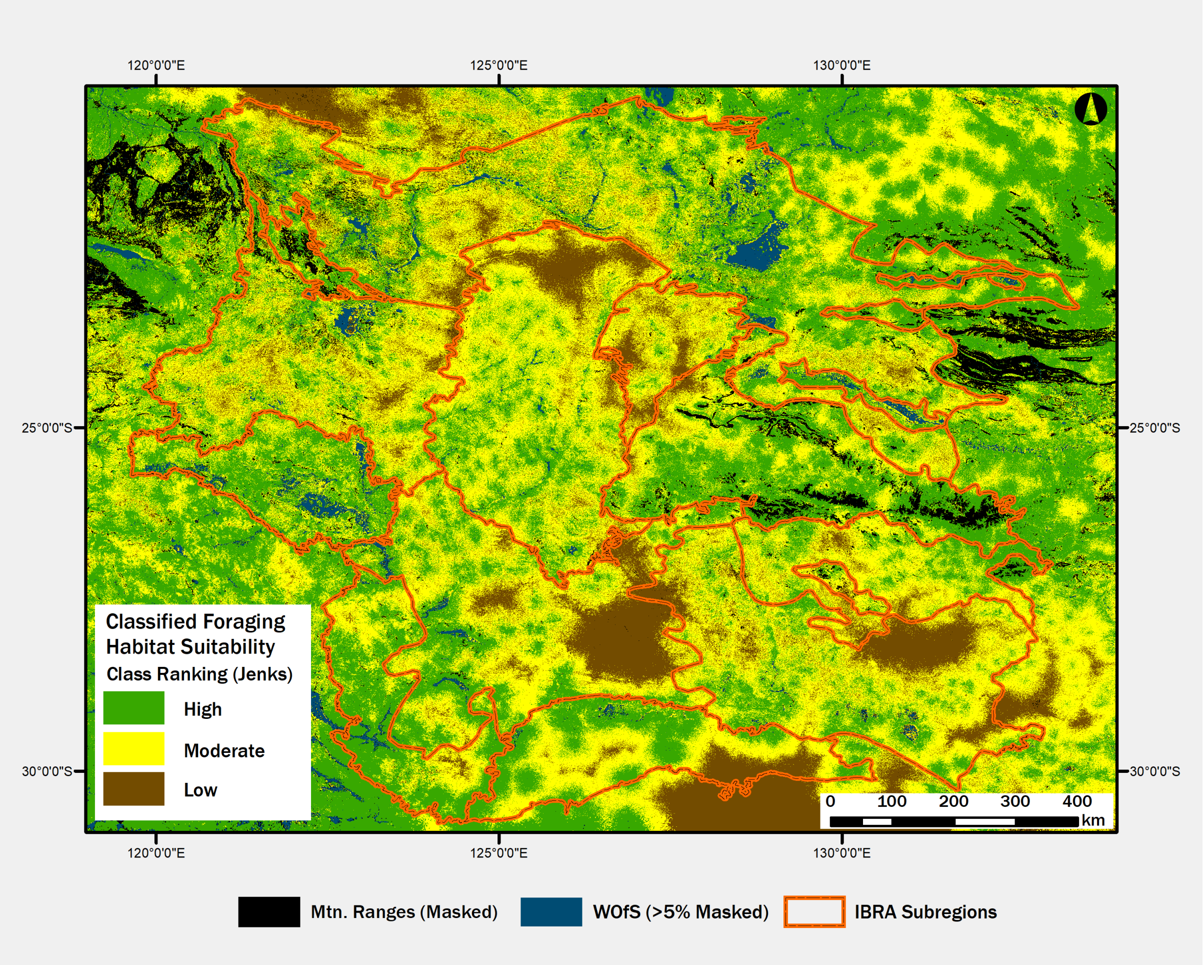

Fig. S4. Classified Foraging Habitat Suitability – The low, moderate and high suitability areas of our raster model area based on the Jenks natural breaks classification method (see Methods). Masked mountain ranges (i.e., refugia), masked WOfS (>5.0%) areas and IBRA subregions indicate omitted areas and bioregional boundaries used for zonal statistic calculations. Map created in ArcGIS Desktop 10.5.1 (https://desktop.arcgis.com).
